# The complexity of dementia development and its comorbidities: The collaborative cross‐mouse population for multivarious tasks approach

**DOI:** 10.1002/ame2.70131

**Published:** 2026-01-13

**Authors:** Osayd Zohud, Iqbal M. Lone, Kareem Midlej, Fuad A. Iraqi

**Affiliations:** ^1^ Department of Clinical Microbiology and Immunology, Gray Faculty of Medicine and Health Sciences Tel‐Aviv University Tel Aviv Israel

**Keywords:** Alzheimer's disease, autoimmune diseases, dementia, neurodegenerative diseases, Parkinson's disease, rheumatoid arthritis

## Abstract

The rising incidence of dementia and associated neurodegenerative disorders poses a growing public health challenge. These conditions have traditionally been studied as isolated central nervous system disorders; however, emerging evidence suggests that broader systemic factors, including chronic inflammation, immune dysregulation, metabolic dysfunction, and genetic susceptibility, may also play a role. This review examines the interconnection between autoimmune diseases and metabolic syndromes in the pathogenesis and exacerbation of neurodegeneration. Conditions such as rheumatoid arthritis, systemic lupus erythematosus, and type 1 diabetes mellitus have been associated with a heightened risk of developing dementia through chronic immune activation, blood–brain barrier disruption, and neuroinflammatory signaling. Similarly, metabolic disorders such as diabesity promote insulin resistance and oxidative stress, accelerating cognitive decline. The review also discusses glaucoma as a neurodegenerative condition with autoimmune features, underscoring the need for expanded classification and treatment strategies. A key focus is the utilization of the Collaborative Cross (CC) mouse model, which enables the study of gene–environment interactions across genetically diverse backgrounds. Findings from CC mice reveal strain‐dependent susceptibility to inflammation, cognitive impairment, and gut–brain axis dysfunction, providing a translational bridge to human variability. This review highlights the importance of integrating precision‐based approaches to dementia research that consider systemic influences. Advancing our understanding of these multiorgan interactions holds potential for designing precision‐based therapeutic approaches to postpone the onset or reduce the incidence of neurodegenerative conditions.

## Graphical abstract

The increasing prevalence of dementia and related neurodegenerative diseases—including Alzheimer's disease, Parkinson's disease, multiple sclerosis, and amyotrophic lateral sclerosis—poses a growing public health challenge. These conditions have traditionally been studied as isolated central nervous system disorders, but emerging evidence points to broader systemic factors, including chronic inflammation, immune dysregulation, metabolic dysfunction, and genetic susceptibility. Diseases such as rheumatoid arthritis, systemic lupus erythematosus, and type 1 diabetes mellitus have been linked to increased risk of dementia through chronic immune activation, blood–brain barrier disruption, and neuroinflammatory signaling. Similarly, metabolic disorders such as diabesity promote insulin resistance and oxidative stress, accelerating cognitive decline. A key focus is the application of the Collaborative Cross (CC) mouse model, which enables the study of gene–environment interactions across genetically diverse backgrounds. Findings from CC mice reveal strain‐dependent susceptibility to inflammation, cognitive impairment, and gut–brain axis dysfunction, providing a translational bridge to human variability. This review emphasizes the importance of integrated, precision‐based approaches to dementia research that account for systemic influences.

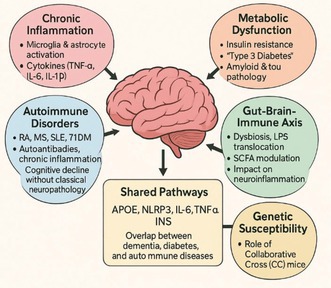

## 1 INTRODUCTION

Neurodegenerative diseases such as Alzheimer's disease (AD), Parkinson's disease (PD), multiple sclerosis (MS), and amyotrophic lateral sclerosis (ALS) are rising rapidly worldwide, with AD alone affecting over 50 million people—a number projected to triple by 2050 due to increased life expectancy and lifestyle changes.^1^ This growing burden underscores the need to identify modifiable risk factors and therapeutic targets.

Traditionally viewed as disorders confined to the central nervous system (CNS), these diseases are now recognized as being influenced by systemic factors, including chronic inflammation, immune dysregulation, and metabolic dysfunction. Pro‐inflammatory mediators, reactive oxygen species (ROS), and immune cell infiltration contribute to neuronal damage and cognitive decline.^2^ Peripheral inflammation, triggered by infections, metabolic disorders, or autoimmunity, can impact the brain through a compromised blood–brain barrier (BBB), which permits immune molecules and cells to enter the CNS and exacerbate pathology. Table 1 summarizes key inflammatory genes such as *JAK–STAT* and *HLA‐DQA1* implicated in these processes. Metabolic disorders—particularly obesity, insulin resistance, and type 2 diabetes mellitus (T2DM)—are also strongly linked to dementia. These conditions impair neuronal metabolism, synaptic function, and neuroprotection, prompting the description of AD as “type 3 diabetes.”^3^

**TABLE 1:** Genetic defects linked to inflammation and autoimmune diseases.

Gene/pathway	Associated disease(s)	Key findings	References
*HLA‐DQA1*, *CTSO*	Sepsis‐associated ARDS	Identified as biomarkers modulating immune responses	[4]
JAK–STAT pathway	Rheumatoid arthritis	Altered gene expression observed in preclinical models	[5]
CISH (cytokine‐inducible SH2‐containing protein)	Autoimmune disorders	Regulates immune checkpoints and cytokine signaling	[6]
Clonal hematopoiesis of indeterminate potential (CHIP) mutations	Autoimmune diseases	Higher CHIP burden associated with increased autoimmune risks	[7]
Immune‐related genes (*SOCS*, *TNF‐α*, *IL‐6*, *IL‐10*, *IL‐1β*)	Various autoimmune diseases	Dysregulated expression correlates with immune dysfunction	[6, 8]
SGLT2 inhibitors (linked genes: *SLC5A2*, *KCNJ11*, *ABCC8*)	Chronic kidney disease (CKD)	Genetic predisposition affects efficacy of these inhibitors	[8]
Mitophagy and oxidative stress–related genes (*PINK1*, *Parkin*, *NLRP3*)	Neuroinflammation, stroke, depression	Links mitochondrial dysfunction to inflammatory responses	[9]
Mast cell inflammatory genes (e.g., *IL‐33*, *TSLP*, *IL‐4R*)	Atopic dermatitis	Genetic predisposition influences inflammatory response in allergic diseases	[10]
SIK2 pathway (SIK2‐CRTC2‐ACC1 axis)	Lipid metabolic disorders, chronic inflammation	Modulates inflammatory response via metabolic regulation	[11]

Abbreviation: ARDS, acute respiratory distress syndrome.

Autoimmune conditions, including MS, systemic lupus erythematosus (SLE), rheumatoid arthritis (RA), and type 1 diabetes mellitus (T1DM), are linked to higher dementia risk. Chronic immune activation and the production of neurotoxic autoantibodies may drive inflammation‐induced neurotoxicity.^12^, ^13^, ^14^, ^15^ Notably, patients with autoimmune disorders often experience cognitive deficits even in the absence of classical neurodegenerative pathology.^16^

To study these complex, systemic contributors to neurodegeneration, genetically diverse animal models are essential. Traditional inbred mouse strains lack the variation found in human populations. Collaborative Cross (CC) mice, a recombinant inbred population, offer a powerful tool for studying gene–environment interactions, including susceptibility to inflammation, metabolic dysfunction, and immune dysregulation.

This review examines the role of chronic inflammation, autoimmune diseases, and metabolic disorders in contributing to neurodegeneration, with a focus on evidence from CC mouse models. Table 2 highlights overlapping genetic contributors, such as apolipoprotein E (APOE), interleukin‐6 (IL‐6), and NLRP3, that span AD, autoimmune, and metabolic pathways, underscoring shared mechanisms driving cognitive decline.

**TABLE 2:** Shared genetic defects in autoimmune diseases, dementia, Alzheimer's disease, and diabetes.

Gene/pathway	Associated diseases	Role in disease mechanism	References
APOE (apolipoprotein E)	Alzheimer's disease, type 2 diabetes, atherosclerosis	Involved in lipid metabolism, neurodegeneration, and insulin resistance	[17]
CETP (cholesteryl ester transfer protein)	Alzheimer's disease, cardiovascular disease, diabetes	Regulates lipid metabolism and inflammation in neurodegeneration	[18]
HLA genes (*HLA‐DQA1*, *HLA‐DRB1*)	Autoimmune diseases (type 1 diabetes [T1D], RA, multiple sclerosis [MS]), Alzheimer's disease	Key regulators of immune responses, linked to neuroinflammation	[17]
TNF‐α (tumor necrosis factor‐α)	Autoimmune diseases, Alzheimer's disease, type 2 diabetes	Pro‐inflammatory cytokine involved in chronic inflammation	[17]
IL‐6 (interleukin‐6)	RA, Alzheimer's disease, type 2 diabetes	Chronic inflammation contributor; regulates immune responses	[18]
IL‐1β (interleukin‐1β)	Autoimmune diseases, Alzheimer's disease, type 2 diabetes	Key mediator of neuroinflammation and β‐cell dysfunction	[17]
*INS* (insulin gene)	Type 1 and type 2 diabetes, Alzheimer's disease	Insulin resistance is linked to neurodegeneration	[18]
*NLRP3* (NLR family pyrin domain containing 3)	Autoimmune diseases, Alzheimer's disease, type 2 diabetes	Regulates inflammasome activation leading to chronic inflammation	[18]
GSK3β (glycogen synthase kinase 3β)	Alzheimer's disease, diabetes, autoimmune diseases	Plays a role in inflammation, neurodegeneration, and insulin signaling	[17]
PTPN22 (protein tyrosine phosphatase nonreceptor type 22)	Type 1 diabetes, RA, Alzheimer's disease	Regulates immune response, associated with chronic inflammation	[18]

Recent advances in cerebral organoid technology (three‐dimensional [3D] cultures derived from induced pluripotent stem cells [iPSCs]) enable modeling of patient‐specific neuronal and glial interactions in vitro.^19^ These organoids provide a scalable tool for studying early and progressive pathological mechanisms in dementia. When combined with the genetic diversity and systemic complexity of CC mice, they create a translationally complementary framework that bridges molecular precision with whole‐organism complexity.^20^

### 1.1 Neuroinflammation in neurodegenerative diseases

Neuroinflammation serves as a key pathological marker of neurodegenerative conditions, contributing to both early pathological changes and disease progression. Although the immune system is strongly implicated in maintaining neural integrity and function, its chronic activation may lead to neurodegeneration, impaired synaptic activity, and progressive cognitive deterioration. In conditions such as AD, PD, ALS, and MS, persistent inflammation in the CNS is driven by pro‐inflammatory cytokines, microglial activation, and oxidative stress.^21^

Microglia, the principal immune surveillance cells within the CNS, are central to this process. Under normal conditions, they facilitate synaptic pruning, neuronal support, and clearance of debris. Nevertheless, overactivation of microglia by injury, infection, or protein aggregation leads to the release of key inflammatory mediators, such as tumor necrosis factor‐α (TNF‐α), IL‐6, and interleukin‐1β (IL‐1β), which impair neuronal function. These cytokines disrupt synaptic transmission and long‐term potentiation (LTP), which are critical for memory and learning, thereby accelerating cognitive decline.

Recent studies using the CC mouse population have demonstrated significant strain‐dependent differences in microglial reactivity, cytokine expression, and BBB disruption. Some CC strains exhibit exaggerated neuroinflammatory responses following systemic challenge, closely resembling the variability in human neurodegenerative susceptibility.^22^, ^23^

Astrocytes, another glial cell type, exacerbate neuroinflammation through reactive gliosis. Under stress, they release glutamate and ROS, leading to excitotoxicity and oxidative injury. Together, microglial and astrocytic responses orchestrate a detrimental inflammatory cascade that undermines neuronal health in neurodegenerative disorders.

Recent evidence suggests that microglial activation is not a binary switch but involves a spectrum of phenotypic states, including the disease‐associated microglia (DAM) profile observed in AD. DAM cells are characterized by upregulated expression of *TREM2*, *APOE*, and lysosomal genes, and are thought to play a dual role in promoting both protective and pathological processes, depending on the disease stage.^24^, ^25^

Furthermore, the cGAS–STING pathway—a cytosolic DNA‐sensing mechanism—has emerged as a novel link between neuroinflammation and cellular senescence. Its activation in microglia and astrocytes triggers sustained type I interferon responses, contributing to neuronal injury.^26^ However, conflicting data suggest that STING inhibition may impair host defense, underscoring the complexity of targeting innate immunity in neurodegenerative settings.

These recent insights suggest that microglial involvement is more complex than previously thought and underscore the need for temporal and cell‐specific targeting in anti‐inflammatory interventions.

### 1.2 Peripheral inflammation and blood–brain barrier dysfunction

Recent studies suggest that peripheral inflammation can directly impact the CNS by compromising the BBB. The BBB represents a selective filter, controlling the trafficking of immune cells, toxins, and metabolic waste from the bloodstream into the brain. However, in long‐standing inflammatory conditions such as obesity, metabolic dysfunction, and autoimmunity, the integrity of the BBB becomes compromised, enabling immune cell migration and cytokine diffusion across it.^27^, ^28^

This peripheral‐CNS inflammatory cross talk is believed to contribute to the underlying mechanisms of multiple neurodegenerative disorders. For example, subjects with chronic immune‐mediated conditions, such as RA, SLE, and inflammatory bowel disease (IBD), exhibit a significantly higher prevalence of neurodegenerative disorders, supporting the link between systemic inflammation and neurodegeneration.^29^

In addition to autoimmune and metabolic inflammation, chronic viral infections are now recognized as potential contributors to neurodegeneration. Herpes simplex virus type 1 (HSV‐1), Epstein–Barr virus (EBV), and cytomegalovirus (CMV) have been associated with long‐term immune activation and an increased risk for AD through latent reactivation, molecular mimicry, and disruption of the BBB integrity. Furthermore, studies following the COVID‐19 pandemic have raised questions about whether SARS‐CoV‐2 infection—and, in some cases, vaccination—may influence neuroimmune pathways relevant to neurodegeneration, although findings remain inconclusive. These emerging data underscore the need for ongoing vigilance regarding both persistent viral antigens and vaccine‐induced immunomodulation as potential modifiers of cognitive trajectories.^30^, ^31^, ^32^, ^33^

### 1.3 Inflammation and protein aggregation in Alzheimer's and Parkinson's disease

Chronic neuroinflammation plays a central role in accelerating the misfolding and aggregation of pathogenic proteins in AD and PD. In AD, amyloid‐β (Aβ) and tau accumulate, whereas in PD, α‐synuclein aggregation contributes to dopaminergic neurodegeneration. Sustained inflammatory signaling—particularly through cytokines such as TNF‐α and IL‐1β—impairs microglial phagocytosis and promotes the transition of astrocytes into a reactive state, which releases excess glutamate and drives excitotoxicity. Activated microglia also exhibit reduced expression of TREM2 and CD33, receptors essential for clearing aggregated peptides, thereby facilitating plaque accumulation and persistent neurotoxicity.^34^, ^35^ This impaired clearance mechanism, together with chronic inflammation, exacerbates protein deposition and neuronal toxicity in both diseases.^36^ In PD specifically, activated microglia release inflammatory cytokines that heighten oxidative stress in the substantia nigra, accelerating dopaminergic neuronal loss and contributing to progressive motor dysfunction.^2^

Emerging evidence suggests that inflammation may modulate proteinopathy via post‐translational modifications (PTMs) of key aggregation‐prone proteins. For example, inflammatory cytokines such as IL‐1β and TNF‐α upregulate kinases like GSK3β and CDK5, which phosphorylate tau, enhancing its propensity to aggregate into neurofibrillary tangles.^37^, ^38^, ^39^

In PD, pro‐inflammatory signaling has been shown to induce nitration and ubiquitination of α‐synuclein, accelerating the formation of Lewy bodies. Notably, activated microglia release reactive nitrogen species that can drive these modifications.^40^ This cross talk between immune mediators and protein PTMs is increasingly viewed as a central driver of neurodegeneration, rather than a secondary consequence.

Cerebral organoids derived from iPSCs have been used to model amyloid and tau pathology in AD. These 3D cultures can recapitulate extracellular Aβ plaque formation, tau hyperphosphorylation, and even glial activation.^20^ Unlike 2D cultures, organoids exhibit structural polarity and synaptic networks that more closely mirror human brain development, making them an ideal system for testing therapeutic targets aimed at reducing aggregation or restoring proteostasis. Recent advances in iPSC‐derived cerebral organoids have enabled researchers to model patient‐specific neuropathological processes in vitro. These 3D, self‐organizing neural cultures closely mimic aspects of early human brain development and have emerged as a powerful tool to study the molecular mechanisms of neurodegenerative diseases, including AD, PD, and frontotemporal dementia (FTD).

When exposed to pro‐inflammatory cytokines or cocultured with activated microglia, cerebral organoids exhibit hallmark features of neurodegeneration, including Aβ plaque accumulation, tau hyperphosphorylation, and neurofibrillary tangle formation, as well as α‐synuclein aggregation. For example, it has been demonstrated that the stimulation of TNF‐α and IL‐1β in iPSC‐derived organoids triggers both synaptic loss and pathological tau conformations, similar to those observed in Braak stage III‐IV profiles. Similarly, CRISPR‐engineered cerebral organoids were used to model familial AD mutations, and they were found to exhibit robust Aβ deposition alongside glial activation and oxidative stress markers.^41^, ^42^, ^43^

Although organoids excel at capturing human‐specific, cell‐autonomous mechanisms, they lack systemic inputs such as vasculature, peripheral immunity, and metabolic signals. In contrast, CC mice offer a genetically diverse, whole‐animal model that can integrate environmental, hormonal, and immune interactions—factors crucial in the progression of dementia.

When used together, organoids and CC mice provide a translationally complementary platform: organoids enable high‐resolution studies of disease initiation and drug response in a human context, whereas CC mice allow for the evaluation of systemic responses, behavioral phenotypes, and long‐term outcomes. This integrated approach enhances mechanistic insight and improves the fidelity of preclinical models for neurodegeneration.

### 1.4 Gut microbiota and inflammation in neurodegeneration

Recent research has highlighted that the gut–brain–immune connection plays a pivotal role in controlling neuroinflammation. The gut microbiome (GM) plays a crucial role in modulating immune system activity, regulating metabolic processes, and influencing neurotransmitter production. Disruptions within the intestinal microbiome, also known as microbial dysbiosis, have been linked to chronic systemic inflammation and an increased risk of neurodegeneration. For instance, patients with IBD and gut dysbiosis have been found to have higher levels of systemic inflammation, elevated BBB leakage, and excessive immune reactivity.^44^ These factors contribute to brain inflammation, accelerating neuronal degeneration in diseases such as AD and PD. Studies using CC mice have demonstrated that different genetic backgrounds affect how gut microbiota imbalances impact neuroinflammation. Certain CC mouse strains exhibit greater resilience to neuroinflammatory changes, suggesting that genetic predisposition plays a role in gut–brain interactions.^45^ These results underscore the importance of investigating individual variability in inflammatory responses and their role in neurodegenerative disease progression.

The CC mouse model has proven instrumental in identifying how host genetics modulate microbiome composition and inflammation‐induced cognitive impairment. For example, it has been demonstrated that CC strains differ markedly in gut barrier integrity, microbial translocation, and downstream neuroimmune activation—highlighting a genetically driven gut–brain communication axis.^46^

The gut–brain axis (GBA) is a bidirectional communication network that involves the GM, the intestinal barrier, systemic immunity, and the BBB. Disruptions in GBA function have been implicated in numerous neurodegenerative and psychiatric disorders, including AD, PD, depression, and MS.^47^

Recent advances in iPSC technology and organ‐on‐a‐chip (OoC) platforms have enabled the modeling of complex GBA interactions under human‐specific conditions. iPSC‐derived intestinal organoids have been shown to reproduce many features of the gut epithelium, including goblet, Paneth, and enteroendocrine cell populations.^48^, ^49^ Cocultures of immune cells and microbial components, such as *Lactobacillus* or *Salmonella*, have been used to study microbial–epithelial interactions, cytokine responses, and epithelial barrier integrity.

Numerous intestinal diseases are characterized by immune activation and disruption of the epithelial barrier. In a seminal study using Caco‐2 epithelial monolayers, it was shown that treatment with pro‐inflammatory cytokines—specifically interferon‐γ (IFN‐γ) and TNF‐α—synergistically induced myosin light‐chain kinase (MLCK) expression and enhanced myosin II regulatory light‐chain (MLC) phosphorylation. These changes led to impaired tight junction morphology, reduced transepithelial electrical resistance (TEER), and increased permeability—hallmarks of the “leaky gut” phenotype. Notably, these effects were reversed by low‐dose sulfasalazine, which prevented TNF‐α‐induced MLCK upregulation and barrier dysfunction independent of NF‐κB inhibition, highlighting a potential therapeutic axis.^50^

Although CC mice offer insight into in vivo gut–brain communication across diverse genetic backgrounds, intestinal organoids and GBA‐on‐a‐chip systems allow for mechanistic dissection of microbial, epithelial, and immune interactions at high resolution. These platforms are especially promising for studying host‐specific variables (e.g., APOE genotype or iPSC‐derived gut models from patient donors) and for screening gut‐targeted therapeutics under human‐relevant conditions.

### 1.5 Potential therapeutic strategies for neuroinflammation

Due to the significant interplay observed between prolonged inflammation and neurodegenerative pathology, several anti‐inflammatory strategies are being explored to mitigate neuroinflammatory damage:

1. Cytokine inhibitors: Monoclonal antibodies targeting pro‐inflammatory cytokines (e.g., TNF‐α blockers, IL‐6 inhibitors) have demonstrated potential in mitigating neuroinflammatory responses in both AD and PD models.^51^, ^52^
2. Microbiome‐based therapies: Probiotic supplementation and fecal microbiota transplants (FMT) are being studied as potential treatments to restore intestinal microbial homeostasis and attenuate systemic inflammation.^53^
3. Dietary interventions: Anti‐inflammatory diets such as the Mediterranean diet have been studied in clinical studies. Clinical studies have demonstrated that adherence to the Mediterranean diet—characterized by high levels of fiber, polyphenols, and omega‐3 fatty acids—can attenuate neuroinflammation and delay cognitive deterioration.^54^
4. Nonsteroidal anti‐inflammatory drugs (NSAIDs): Findings from population‐based studies suggest that chronic NSAID administration may correlate with a decreased likelihood of developing AD. Nevertheless, clinical trial outcomes have been inconsistent, indicating the need for further investigation into timing and patient‐specific factors.^55^
5. Metabolic regulation: Drugs such as GLP‐1 receptor agonists (e.g., liraglutide), initially designed as a therapeutic agent to manage diabetic conditions, have shown neuroprotective actions involving the downregulation of pro‐inflammatory pathways, ROS, and insulin resistance in the brain.^56^

### 1.6 The gut–brain–immune axis and neurodegeneration

The intestinal microbiome plays a crucial role in regulating immune responses and brain health. Growing scientific findings suggest that dysregulated gut microbiota can contribute to neurodegeneration through inflammatory and metabolic pathways.^57^ Disruptions in the intestinal microbiome can increase gut permeability (leaky gut), leading to bacterial components such as endotoxins and lipopolysaccharides (LPS) to access the circulatory system. These endotoxins trigger systemic immune activation and are capable of penetrating the BBB, promoting neuroinflammation.^58^, ^59^

### 1.7 Gut microbiota and neurodegeneration in CC mice

Emerging work in CC mice demonstrates that host genetics plays a decisive role in shaping gut microbial communities and, consequently, modulating susceptibility to neuroinflammatory and neurodegenerative processes. Across CC strains, substantial variation has been observed in baseline microbial diversity, immune tone, and gut–brain signaling pathways. Notably, certain strains exhibit marked resistance to gut dysbiosis–induced neuroinflammation, suggesting that protective genetic architectures can buffer the CNS against microbe‐derived inflammatory cues.^44^ These findings underscore the importance of gene–environment interactions in determining how disturbances in the gastrointestinal microbiome influence microglial activation, cytokine expression, and downstream neuronal vulnerability. CC mice, therefore, offer a uniquely powerful platform for dissecting the causal mechanisms linking gut microbiota composition to neurodegenerative phenotypes.^44^

Findings from CC mice can be further complemented by brain organoid models, which allow controlled manipulation of microbial metabolites and inflammatory cues to dissect the cell‐intrinsic pathways underlying microglial and neuronal responses. Although organoids lack the full complexity of the GBA, they offer a reductionist platform for mechanistic validation of microbiome‐driven neuroinflammatory signals.

## 2 DIABETES, INSULIN RESISTANCE, AND ALZHEIMER'S DISEASE: “TYPE 3 DIABETES”?

In the past 20 years, researchers have identified a significant connection linking metabolic disorders and neurodegeneration, particularly AD. A growing body of evidence has confirmed that insulin resistance, chronic hyperglycemia, and obesity are significantly implicated in the increased incidence of cognitive deterioration.^2^ This has led to the concept of AD as “type 3 diabetes,” emphasizing the contribution of metabolic disturbances to neurodegenerative processes.

### 2.1 The role of insulin in the brain

The regulatory function of insulin is well established in glucose metabolism, but it is additionally essential for maintaining neurological health. Neurons require insulin to regulate synaptic plasticity, neurotransmitter balance, and energy metabolism. Insulin signaling in the brain influences LTP, a fundamental mechanism underlying learning and memory.^60^ However, in subjects with T2DM or metabolic syndrome, insulin resistance develops, impairing glucose uptake in neurons. As a result, brain cells experience energy deficits, increased oxidative stress, and mitochondrial dysfunction, all of which contribute to synaptic failure and cognitive impairment.^61^

### 2.2 Brain glucose hypometabolism in Alzheimer's disease

A hallmark of AD is glucose hypometabolism, in which neurons fail to utilize glucose efficiently. Functional imaging studies consistently demonstrate reduced glucose uptake in key memory‐associated regions, including the hippocampus and prefrontal cortex.^51^, ^52^ This metabolic deficit is closely linked to insulin resistance, which interferes with the clearance of Aβ peptides. In normal physiology, the insulin‐degrading enzyme (IDE) helps metabolize Aβ; however, insulin‐resistant states impair IDE activity, allowing amyloid peptides to accumulate and form toxic extracellular plaques. These disruptions in glucose handling and Aβ clearance set the stage for widespread synaptic dysfunction and early neurodegenerative changes in AD.

At the molecular level, AD‐related insulin resistance impairs insulin receptor substrate‐1 (IRS‐1) signaling, leading to defective AKT phosphorylation, reduced expression of the glucose transporter 4 (GLUT4), and heightened activation of glycogen synthase kinase 3β (GSK3β). These alterations weaken synaptic plasticity and LTP, two processes essential for learning and memory.^62^, ^63^ Dysregulated GSK3β activity further contributes to tau hyperphosphorylation, driving the formation of neurofibrillary tangles that destabilize neuronal architecture, impair axonal transport, and accelerate cognitive decline 3. Together, these metabolic and signaling abnormalities highlight how insulin resistance amplifies both amyloid and tau pathology in AD.^64^

Insights from genetically diverse CC mice further underscore the influence of host genetics on metabolic vulnerability. Certain CC lines exhibit marked differences in baseline glucose handling, mitochondrial efficiency, and insulin‐signaling responsiveness, traits that modulate susceptibility to neurodegeneration under metabolic stress.^45^ These strain‐specific metabolic phenotypes suggest that genetic background strongly shapes how impaired insulin signaling contributes to AD‐related pathology.

Complementing these in vivo findings, iPSC‐derived cortical organoids generated from individuals with insulin resistance or APOE4 variants exhibit impaired glucose uptake and mitochondrial dysfunction. These models enable targeted interrogation of insulin–PI3K–AKT signaling and provide a scalable platform for evaluating metabolic enhancers or mitochondrial‐targeted therapeutics in early Alzheimer's contexts.^65^ When integrated with CC mouse data, organoid systems offer a powerful cross‐model approach to dissecting how genetic background, cellular metabolism, and insulin signaling converge to drive AD‐related neurodegeneration.

### 2.3 Cerebral organoid models of metabolic dysfunction: Recapitulating obesity‐, diabetes‐, or lipid‐toxicity‐induced brain vulnerability in vitro

Recent advances in human cerebral organoid technology have opened new avenues for studying the molecular impact of metabolic dysfunction on the brain. These self‐organizing, stem cell–derived 3D cultures recapitulate key features of brain development and are particularly useful for dissecting the cellular effects of insulin resistance, hyperglycemia, and lipotoxicity.

In vitro exposure of cerebral organoids to diabetic conditions—such as high‐glucose, high‐fatty acid media—has been shown to induce neuronal oxidative stress, mitochondrial dysfunction, and synaptic protein loss, mimicking early Alzheimer's‐like pathology. A recent study demonstrated that prolonged exposure to palmitate or advanced glycation end‐products (AGEs) in iPSC‐derived organoids led to increased expression of pro‐inflammatory cytokines (e.g., IL‐1β, TNF‐α) and tau hyperphosphorylation.^66^

These models also respond to antidiabetic treatments. Organoids treated with GLP‐1 receptor agonists exhibit enhanced neuronal insulin signaling and reduced amyloidogenic processing, indicating that cerebral organoids can serve as a platform for testing neuroprotective metabolic interventions.^67^, ^68^, ^69^ Moreover, organoids derived from individuals with metabolic syndrome exhibit patient‐specific vulnerabilities to lipotoxic stress, reinforcing their utility in personalized medicine.^70^

Although CC mouse models provide insight into systemic and genetic interactions, cerebral organoids complement these findings by revealing cell‐intrinsic mechanisms under human‐specific conditions. Integrating both models enables a multiscale understanding of how metabolic disorders contribute to neurodegeneration.

### 2.4 Diabetes‐associated vascular damage and neurodegeneration

Diabetes and chronic hyperglycemia contribute to BBB dysfunction and cerebrovascular damage, further exacerbating neurodegeneration. Diabetes‐associated vascular complications, including microvascular damage, endothelial dysfunction, and chronic inflammation, disrupt cerebral perfusion, thereby limiting oxygen and nutrient supply to neurons.^57^

Strain‐specific CC mouse studies have revealed distinct patterns of brain glucose metabolism and insulin signaling, offering insights into genetic susceptibilities to Alzheimer's‐like glucose hypometabolism. These models allow dissecting the genetic architecture behind insulin resistance–linked neurodegeneration.^45^

AGEs accumulation in diabetes fuels oxidative and inflammatory stress, worsening neuronal degeneration.^71^ Diabetic individuals often present heightened concentrations of pro‐inflammatory cytokines, including TNF‐α, IL‐6, and IL‐1β, which contribute to both peripheral and central neuroinflammation.

Given the strong association between diabetes, insulin resistance, and cognitive decline, several therapeutic strategies are being investigated:

1. Metformin: A widely used antidiabetic drug, metformin has neuroprotective properties through the attenuation of oxidative stress, inflammation, and cognitive decline in AD models.^72^
2. GLP‐1 receptor agonists (liraglutide, semaglutide): These medications, initially developed for diabetes, have demonstrated significant neuroprotective properties in both preclinical and clinical trials. GLP‐1 agonists enhance neuronal insulin signaling, decrease amyloid burden, and reduce neuroinflammation.
3. Ketogenic diet and caloric restriction: Low‐carbohydrate diets that promote ketone metabolism have been explored as potential interventions to improve brain energy metabolism and reduce neuroinflammation.^54^
4. SGLT2 inhibitors: These drugs, designed to lower blood sugar, have shown promising results in reducing oxidative stress and inflammation, potentially protecting against cognitive impairment.^53^

## 3 AUTOIMMUNE DYSFUNCTION IN NEURODEGENERATIVE DISORDERS

In recent years, autoimmune diseases have emerged as significant risk factors for neurodegeneration, with numerous studies demonstrating a well‐established association with chronic immune activation and cognitive impairment. Immune‐mediated diseases such as MS, RA, SLE, and T1DM have been linked to a heightened likelihood of onset of AD and PD.^51^, ^52^

### 3.1 How autoimmune dysregulation contributes to neurodegeneration

Autoimmune diseases are characterized by dysregulated immune responses, marked by self‐directed immune activity against the body's own cells. In neurodegenerative conditions, this autoimmune dysfunction contributes to chronic neuroinflammation, BBB disruption, and neurotoxicity.^21^ Patients with RA and SLE exhibit higher concentrations of pro‐inflammatory mediators in circulation, which contribute to neuronal damage and cognitive decline.^2^ Furthermore, numerous studies have identified autoantibodies targeting neuronal proteins in patients with neurodegenerative and autoimmune diseases. For example, anti‐NMDA receptor antibodies have been linked to cognitive dysfunction in lupus, whereas anti–α‐synuclein antibodies are detectable in patients with PD and may influence dopaminergic neuron loss. These autoantibodies can disrupt synaptic transmission, trigger complement‐mediated neurotoxicity, and may cross‐react with self‐antigens due to molecular mimicry.^57^

### 3.2 Multiple sclerosis and neurodegeneration

MS is a prototypical neuroimmune disorder, in which autoreactive T cells attack the myelin sheath surrounding neurons, leading to chronic inflammation, axonal damage, and cognitive impairment.^12^ Interestingly, findings from recent investigations suggest that MS patients exhibit a markedly elevated risk of developing AD, reinforcing the autoimmune–neurodegeneration connection.^73^, ^74^, ^75^ Dysregulated T‐cell and B‐cell responses in MS contribute to chronic CNS inflammation, which accelerates synaptic loss and neuronal degeneration. In experimental autoimmune encephalomyelitis (EAE) models based on the CC population, strain‐specific immune responses have been shown to correlate with differential CNS infiltration and demyelination, aiding in mapping neuroimmune loci.^76^

### 3.3 Autoimmune‐linked cognitive decline in Alzheimer's and Parkinson's diseases

Patients with RA, SLE, and T1DM often exhibit cognitive impairment, even in the absence of classical neurodegenerative pathology. Elevated pro‐inflammatory markers in these conditions suggest a shared inflammatory pathway between autoimmune diseases and neurodegenerative disorders.^54^ In PD, autoantibodies against α‐synuclein have been identified, suggesting that autoimmune mechanisms contribute to dopaminergic neuron loss.^2^ Additionally, individuals with chronic autoimmune inflammation exhibit accelerated aging‐related neurodegeneration, supporting the role of long‐term immune dysregulation in cognitive decline.

### 3.4 Potential autoimmune therapeutic strategies for neurodegeneration

Given the strong overlap between autoimmunity and neurodegeneration, researchers are exploring immune‐modulating therapies as potential treatments:

1. Monoclonal antibodies (e.g., anti‐CD20, rituximab): Originally used for MS and RA, these therapies are now being investigated for their capacity to attenuate neuroinflammation in AD and PD.^53^
2. Cytokine inhibitors (e.g., TNF‐α and IL‐6 blockers): These drugs may reduce chronic neuroinflammation and slow cognitive decline.^22^
3. Gut microbiome modulation: Autoimmune diseases are often associated with gut dysbiosis, and interventions such as probiotics and FMT are being explored to restore immune balance and reduce neuroinflammation.^57^

### 3.5 Sex differences in neurodegeneration and autoimmunity

Sex hormones significantly influence immune regulation and may partly explain sex‐based differences in the incidence and development of neurodegenerative and autoimmune disorders. Estrogen, in particular, exhibits anti‐inflammatory characteristics that contribute to its neuroprotective effects. This may help explain why premenopausal women exhibit a reduced risk of AD relative to age‐matched men.^77^ Furthermore, women are disproportionately affected by autoimmune conditions, including MS and SLE, both of which are tied to a heightened likelihood of developing cognitive decline and dementia.^54^ These observations suggest that hormonal differences and immune system dynamics play a central role in shaping susceptibility to neurodegeneration across sexes.

### 3.6 Environmental and lifestyle factors in neurodegeneration

Neurodegenerative disease susceptibility is substantially affected by environmental and behavioral factors. Diets high in saturated fats and sugars can raise chronic inflammation, whereas Mediterranean diets—rich in fiber, polyphenols, and omega‐3 fatty acids—support gut and brain health.^78^ Regular exercise reduces neuroinflammation, improves insulin sensitivity, and enhances cognitive function.^79^ Air pollution is also a growing concern, with evidence linking long‐term exposure to an increased risk of AD through oxidative stress and brain inflammation.^80^ These findings underscore the significance of modifiable behaviors in mitigating cognitive decline.

## 4 IMMUNE CROSS TALK IN RA, DIABETES, AND ALZHEIMER'S DISEASE

Autoimmune diseases such as RA and psoriasis are increasingly recognized as systemic disorders with far‐reaching implications, including heightened susceptibility to neurodegenerative conditions and metabolic syndromes like diabesity. Chronic low‐grade inflammation, mediated by cytokines such as IL‐6, TNF‐α, and IL‐1β, serves as a shared mechanism linking peripheral autoimmune activity to central neuroinflammatory cascades.^81^ Moreover, aging exacerbates these connections through immunosenescence and reduced immune tolerance, contributing to cognitive decline.^82^ These overlapping mechanisms underscore the importance of comprehensive therapeutic strategies that target inflammatory processes across multiple organ systems and life stages.

Recent integrative models of chronic disease point to the immune system as a central node linking autoimmune conditions such as RA with both type 2 diabetes (T2D) and progressive neurological conditions, for instance, AD. Doroszkiewicz et al.^83^ propose a model of innate immune dysregulation in which inflammatory triggers, particularly the cytokine storm involving TNF‐α, IL‐6, and c‐reactive protein (CRP), lead to insulin receptor desensitization. They highlight α2‐macroglobulin (α2M)—a key protease inhibitor elevated in RA and T2D—as a molecule that impairs clearance of Aβ, further linking immune dysfunction with neurotoxicity. Notably, this same immune signature is implicated in IBD, reinforcing the concept of common inflammatory circuitry across organ systems. As such, autoimmune pathology may no longer be seen as organ‐specific but rather as part of a systemic process that accelerates both metabolic syndrome and cognitive deterioration.^83^

### 4.1 Insulin resistance and neurodegeneration: Molecular pathways

Insulin resistance, although typically discussed in the context of metabolic disease, has emerged as a neuropathological hallmark of AD. Alves et al.^84^ provide a compelling synthesis of how insulin signaling deficits disrupt downstream pathways essential for neuronal survival, including those regulating tau phosphorylation, amyloid precursor protein processing, and mitochondrial oxidative balance. Their analysis suggests that autoimmune‐driven chronic inflammation, as observed in RA, may act as a primer for central insulin resistance through the upregulation of cytokine‐induced SOCS3 and serine phosphorylation of IRS‐1. These pathways impair synaptic plasticity and memory formation, suggesting that autoimmune inflammation may be a covert driver of cognitive aging. Importantly, RA therapies targeting TNF‐α and IL‐1β may also mitigate neurodegenerative risk by restoring insulin pathway sensitivity.^84^

### 4.2 Systemic inflammation and cognitive dysfunction in RA

Neurocognitive symptoms in patients with RA have traditionally been attributed to psychosocial stress and chronic pain. However, Basile et al.^85^ provide molecular evidence that RA itself may induce direct neuroinflammatory effects. Through the chronic secretion of IL‐1β, TNF‐α, and IL‐17, RA promotes microglial activation, oxidative stress, and impairment of BBB permeability and protective function. These effects culminate in disrupted synaptic homeostasis and reduced hippocampal neurogenesis—hallmarks of early cognitive decline. The authors also link systemic inflammation to impaired autophagic clearance of misfolded proteins, a mechanism relevant both in RA joint pathology and AD. Thus, cognitive dysfunction in RA may not be incidental but instead represents a direct consequence of immune‐mediated neurodegeneration.

### 4.3 Repositioning antidiabetic drugs for neuroprotection

In light of the shared inflammatory and insulin‐resistant states across RA, T2D, and AD, there is growing interest in repurposing antidiabetic drugs for cognitive benefit. Mantik et al.^86^ review a range of antidiabetic agents, particularly metformin and pioglitazone, which have demonstrated the ability to enhance mitochondrial efficiency, attenuate oxidative damage, and restore insulin receptor sensitivity in both peripheral tissues and the brain. These drugs not only modulate glucose metabolism but also appear to inhibit NF‐κB signaling, a pathway shared in RA and AD. The study posits that targeting peripheral insulin resistance could indirectly ameliorate central neuroinflammatory processes, offering a dual benefit in autoimmune‐metabolic syndromes.

### 4.4 Intranasal insulin: Targeting brain insulin resistance

A more targeted approach to address central insulin resistance has emerged in the form of intranasal insulin therapy. Craft et al.^87^ conducted a randomized controlled investigation in subjects with mild cognitive impairment and AD, finding that intranasal insulin delivery improved verbal memory and executive function without systemic side effects. This method bypasses the BBB, directly enhancing central insulin availability and signaling. Given that RA patients often present with insulin resistance and subtle cognitive impairments, this strategy may hold potential as a cross‐domain intervention that alleviates both metabolic and cognitive symptoms in chronic inflammatory states.^88^ Although long‐term safety and efficacy require further investigation, these initial results are promising for populations with multimorbidity.^87^, ^88^

### 4.5 Psoriasis and advanced glycation end‐products: Inflammation across the lifespan

Psoriasis, long regarded as a skin‐limited autoimmune disorder, is increasingly recognized as a systemic inflammatory disease with strong ties to metabolic and neurodegenerative disorders. A pivotal study by Puig and López‐Ferrer demonstrates that patients with moderate‐to‐severe psoriasis exhibit elevated levels of AGEs—biomarkers of oxidative stress and metabolic dysregulation. AGEs are known to cross‐link collagen, activate RAGE receptors, and stimulate NF‐κB‐mediated inflammation, linking psoriasis to insulin resistance, vascular dysfunction, and potentially cognitive decline. The accumulation of AGEs accelerates immunosenescence, making them a significant contributor to both the progression of aging‐related diseases and the severity of psoriatic flares. These findings position psoriasis within a broader inflammatory‐oxidative axis that includes diabetes, atherosclerosis, and neurodegeneration, reinforcing the idea that systemic metabolic inflammation underlies a continuum of chronic diseases.

### 4.6 Mitochondrial dysfunction in psoriasis: Fueling neurodegeneration and diabesity

Mitochondrial health is emerging as a central determinant of chronic disease risk, and recent research by Palmer et al.^89^ underscores how psoriasis disrupts mitochondrial function, linking it to broader metabolic and neurological consequences. The authors detail how chronic cutaneous inflammation induces mitochondrial oxidative stress, reduces ATP production, and impairs cellular redox balance—biochemical features also observed in insulin resistance, AD, and aging tissues. Notably, mitochondrial dysfunction promotes endothelial damage and neurovascular dysregulation, mechanisms that mediate both cardiovascular disease and neurodegeneration. Psoriasis, therefore, cannot be considered in isolation but rather as part of a systemic pathology involving metabolic‐skin‐brain cross talk. These insights underscore the importance of therapeutic approaches that target mitochondrial resilience rather than solely focusing on immune suppression.

### 4.7 Biochemical pathways in psoriasis: A convergent model of metabolic and cognitive disease

The biochemical overlap between psoriasis, diabesity, and neurodegeneration has garnered substantial interest in contemporary scientific investigations. In a systems‐level review, it highlights the shared molecular pathways underlying these comorbidities—particularly the dysregulation of PI3K/AKT/mTOR signaling, insulin receptor desensitization, and chronic cytokine activation. The study notes that psoriatic patients often exhibit increased visceral fat, elevated IL‐6 and TNF‐α, and impaired glucose metabolism—hallmarks of the metabolic syndrome. These molecular patterns overlap with early neuroinflammatory changes, suggesting that psoriasis may act as a sentinel for deeper systemic dysfunction affecting the brain, liver, and pancreas. This integrative perspective supports redefining psoriasis as a multiorgan sentinel disease, calling for coordinated intervention strategies that span dermatological, metabolic, and neurological domains.^90^

## 5 GLAUCOMA

Glaucoma is a common neurodegenerative eye condition characterized by the degeneration of nerve axons and retinal ganglion cells (RGCs). The etiology of glaucoma involves a number of clinically validated risk determinants, including excessive myopia, concomitant conditions such as hypertension and cardiovascular illnesses, elevated intraocular pressure (IOP), and family history.^91^, ^92^ Due to its role as the principal determinant of risk, IOP is a prime candidate for both medicinal and surgical management. Yet, patients with normal IOP can also experience the neurodegenerative process, which can worsen even after current therapeutic approaches have brought IOP back to normal.^93^ Neurological injury has been attributed to several pathogenic processes, including glutamate‐mediated neurotoxicity, nitric oxide (NO), and dysregulation of neuroprotective and neuroinflammatory pathways. Autoimmunity has been implicated in the development of several neurological illnesses in recent decades, and as a result, it has been proposed as a plausible explanation for glaucoma.^94^ The well‐characterized autoimmune disorder RA, which shares multiple autoantigens with glaucoma, is actually more common among glaucoma patients.^95^

### 5.1 Glaucoma, autoimmunity, and heat shock proteins

As previously noted, autoimmune mechanisms play a central role in the pathogenesis of glaucoma, particularly in the degeneration of RGCs. This is evidenced by T‐cell infiltration in the affected retina, the prevention of neurodegeneration in experimental mouse models following the depletion of B cells—and more significantly, T cells—and findings that heat shock protein (HSP)‐derived peptides serve as target antigens for both antibody production and CD4+ T‐cell responses in both serum and retinal tissue.^96^ Furthermore, glaucoma‐like neuropathology could be induced in rats vaccinated with an HSP peptide, and increased IOP was associated with increased HSP expression in retinal tissue. Earlier research in a rat model of cyclooxygenase‐induced ischemia retinal damage (cox‐2) indicated that HSPs may play a part. The HSP‐70i model appeared to have a protective effect. Therefore, antibodies that block HSPs may also play a role in glaucoma‐related retinal damage.

### 5.2 Retinal organoids: A window into glaucoma pathogenesis

Retinal organoids derived from human‐ iPSCs offer an advanced in vitro system for modeling the cellular and molecular events underlying glaucoma‐related neurodegeneration. These 3D structures recapitulate the laminar organization of the retina, including functional RGCs, photoreceptors, and supporting glia.^97^

Recent work using retinal organoids has demonstrated that oxidative stress, mechanical stretch, or HSP stimulation can reproduce early degenerative changes observed in glaucoma patients, including RGC apoptosis and reactive gliosis.^98^ Moreover, retinal organoids can express glaucoma‐linked genes (e.g., *MYOC*, *OPTN*, *HSP70*) and facilitate CRISPR‐based disease modeling, allowing for the mechanistic dissection of gene–environment interactions under human‐like conditions.

Importantly, organoids exposed to pro‐inflammatory cytokines (e.g., IL‐1β, TNF‐α) develop axon degeneration and synaptic disruption, mirroring neuroinflammatory processes also seen in CC mouse models. Some protocols have begun to integrate retinal organoids with microglia‐like cells, advancing their utility for studying immune‐mediated neurodegeneration.^98^, ^99^

This model complements in vivo data by providing a controlled system for studying retinal pathology at early, preclinical stages and facilitating high‐throughput screening of neuroprotective or immunomodulatory compounds. Together with CC mice, retinal organoids can help define shared immune targets and therapeutic candidates across autoimmune glaucoma and CNS neurodegeneration.

### 5.3 Therapeutic implications

Currently, glaucoma treatment focuses solely on the disease's causes, primarily lowering intraocular pressure. There is essentially no information on how to treat degenerative loss of retinal ganglion cells. The information discussed above suggests an immunomodulatory strategy, specifically general‐ or antigen‐specific (HSP‐specific) immunosuppression. One should use proven treatment drugs from RA for glaucoma due to shared immunopathogenic mechanisms among the two conditions, for example, the noticeable infiltration of affected organs by CD4 T cells that target HSPs. Additionally, it is advisable to consider novel interventional approaches like microbiome modification or the application of other microbial metabolites, including short‐chain fatty acids (SCFA). According to recent research by Chen et al.^96^ resident gut microbiota‐induced T lymphocytes play a role in mediating glaucoma neurodegeneration. Many neurodegenerative illnesses, including MS^100^, ^101^ and ALS,^102^ have already been treated or studied with microbiome‐directed therapy.

## 6 COLLABORATIVE CROSS MICE IN NEURODEGENERATIVE RESEARCH

Traditional inbred mouse models have long been used to study neurodegenerative pathologies, such as AD, PD, and MS, and diabetes‐associated cognitive decline. However, these models lack genetic diversity, which limits their ability to accurately reflect the genetic diversity observed in human cohorts. To address this gap, the CC mouse model was designed using eight genetically diverse founder strains, enabling scientists to explore interactions between genetic and environmental factors with greater relevance to human biology.^103^ As shown in Table 3, several genes have been identified as contributors to distinct dementia phenotypes through disruption of lysosomal, mitochondrial, and synaptic pathways.

**TABLE 3:** Genetic defects linked to dementia.

Gene	Associated dementia type	Pathway affected	References
*SORL1*	Alzheimer's disease (AD)	Endolysosomal pathway	[104]
*ADCY3*	AD	Neurodegeneration, ciliary deterioration	[105]
*PLD3*	AD	Lysosomal biogenesis	[106]
*TRPM7*	Parkinsonism‐dementia‐amyotrophic lateral sclerosis (ALS)	Neurodegeneration	[107]
*GBA1*	Lewy body dementia and Parkinson's disease (PD)	Lipid metabolism, lysosomal dysfunction	[108]
*GRN* (progranulin)	Frontotemporal dementia	Lysosomal dysfunction, immune response	[109]
*TDP‐43*	AD and ALS	Cholesterol metabolism, immune response	[110]
α‐Synuclein (*SNCA*)	Parkinson's dementia, Guam PD	Mitochondrial dysfunction	[111]
*eEF2*	Down syndrome‐associated dementia	Synaptic failure, cognitive function	[112]
*APP*, *PSEN1*, *PSEN2*	Early‐onset AD	Amyloid plaque formation	[113]

CC mice serve as a crucial model for examining how genetic variation influences cognitive deficits associated with obesity and diabetes—both known risk factors for neurodegeneration.^45^, ^114^ Studies have demonstrated strain‐specific variability in cognitive decline due to metabolic dysfunction, with some strains exhibiting severe insulin resistance and cognitive deficits, whereas others remain resilient.^45^

In the context of neuroinflammation, CC mice show distinct inflammatory responses. Some strains display elevated levels of TNF‐α, IL‐6, and IL‐1β, increased BBB permeability, and microglial activation—hallmarks of accelerated neurodegeneration in human AD and PD.^2^ Conversely, other strains exhibit resistance, underscoring the genetic basis of inflammatory vulnerability.

CC mice have also shed light on gut–brain interactions in neurodegeneration. Gut dysbiosis in certain strains correlates with increased Aβ accumulation and neuroinflammation, whereas others maintain microbial balance and cognitive function.^44^ These models also help examine variable responses to dietary interventions and microbiome‐based therapies.

In studies of autoimmune disorders such as MS, RA, SLE, and T1DM—all linked to increased dementia risk—CC mice help clarify genetic contributions to immune dysregulation and CNS infiltration. Certain strains develop severe MS‐like pathology, aiding in the identification of neuroimmune regulatory genes.^29^

The association shared by insulin resistance and AD (“type 3 diabetes”) is another area enriched by CC models. Strain‐dependent differences in insulin signaling, glucose metabolism, and Aβ aggregation have been observed, providing insights into genetic susceptibilities to metabolic‐cognitive dysfunction.^45^

Looking forward, CC mice offer promising avenues for identifying genetic profiles linked to neurodegenerative resilience or risk. Genome‐wide association studies (GWAS) using these models may uncover targets for precision medicine.^115^, ^116^ Additionally, CC strains have shown sex‐specific responses to metabolic and inflammatory stressors, making them ideal for exploring hormonal influences on cognitive aging.^77^, ^117^

**FIGURE 1:**
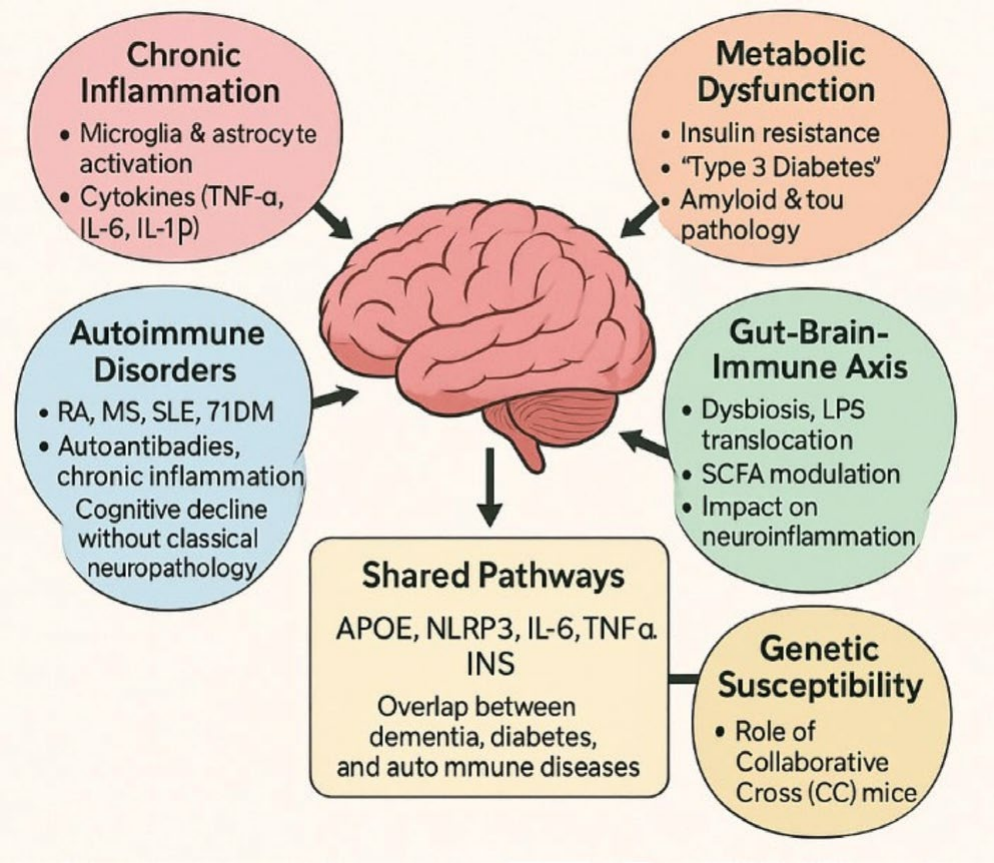
Schematic diagram showing the overall view of the present study.

The capacity of CC mice to reflect individualized therapeutic responses also supports their use in testing personalized interventions ranging from anti‐inflammatory agents and dietary therapies to microbiota modulation.^118^, ^119^ As summarized in Table 4 and illustrated in Figure 1, studies using CC mice have shed light on the roles of genes such as *SORL1* and *ADCY3* in dementia, thereby advancing both our understanding and therapeutic toolkit.

**TABLE 4:** Recent studies on genetic factors in dementia.

Study title	Key findings	Year	References
Investigating the role of *SORL1* in AD‐specific endolysosomal phenotypes	Identifies *SORL1* mutation as a driver of neuronal defects in AD	2025	[104]
Deterioration of neuronal primary cilia in Alzheimer's disease	Finds *ADCY3* as a critical gene in ciliary degeneration linked to AD	2024	[105]
Phospholipase D3 (*PLD3*) regulates lysosomal biogenesis	*PLD3* mutations associated with increased AD risk	2024	[106]
A comprehensive review on androgen deprivation therapy and Alzheimer's disease	Examines androgen therapy's impact on dementia risk	2024	[120]
Role of *TDP‐43* in cholesterol metabolism and implications for AD	Links *TDP‐43* mutations to cholesterol imbalance and AD risk	2024	[110]
Suppression of eEF2 phosphorylation alleviates cognitive deficits in Down's syndrome	Suggests a genetic basis for Down syndrome–associated dementia	2024	[112]
Impact of APOE ε4 in Alzheimer's disease	Significant association of *APOE ε4* allele with Alzheimer's risk and disease progression	2024	[121]
Genome‐wide association meta‐analysis of all‐cause and vascular dementia	Identified multiple genetic variants influencing all‐cause dementia (ACD) and vascular dementia (VaD)	2024	[122]
Exploring genetic predisposition to Alzheimer's: *TOMM40* and *CD33* polymorphisms	Investigates polymorphisms in *TOMM40* and *CD33*, showing their impact on Alzheimer's risk	2024	[123]
Causal relationship between type 2 diabetes and dementia	Found a genetic link between T2DM and Alzheimer's disease, using Mendelian randomization	2025	[3]
Gene expression changes in Alzheimer's hippocampus	Identified 39 differentially expressed genes linked to AD progression	2025	[124, 125]
Brain and blood transcriptome‐wide association studies	Found three novel genes associated with cognitive resilience in AD	2024	[125]
Sex differences in aging and dementia risk	Meta‐analysis showed higher dementia incidence in women due to genetic and hormonal factors	2025	[126]
High BMI and vascular dementia	Found a U‐shaped relationship between BMI and dementia risk, with genetic implications	2024	[127]
Apolipoprotein E ε4 and Alzheimer's disease	Meta‐analysis of voxel‐based morphometry studies confirms *APOE ε4* as a major genetic risk factor	2024	[121]
Inflammatory bowel disease (IBD) and dementia	Identifies a genetic overlap between IBD and dementia using observational and genetic studies	2025	[56]
Genome‐wide association study for dementia with Lewy bodies (DLB)	Discovered an East Asian–specific genetic locus associated with DLB	2025	[128]
Efficacy of acetylcholinesterase inhibitors (AChEIs) in dementia	Systematic review on AChEIs showing reduced hippocampal atrophy in dementia patients	2025	[129]
Retinal imaging biomarkers for dementia	Found genetic markers linking cerebrovascular diseases to dementia via ocular imaging	2024	[130]
Cross‐linking neuropsychiatric symptoms with dementia	Meta‐analysis on genetic and environmental factors influencing neuropsychiatric symptoms in ADRD	2024	[131]

Abbreviations: ARDS, acute respiratory distress syndrome; BMI, body mass index.

## 7 ORGANOID AND CC MOUSE MODELS: COMPLEMENTARY ROLES IN NEURODEGENERATION RESEARCH

Organoid and CC mouse models offer complementary insights into the pathogenesis and treatment of neurodegenerative diseases. Organoids, derived from human iPSCs, capture human‐specific developmental and pathological features, including tau phosphorylation, amyloid precursor processing, and cytokine‐induced neuronal injury under metabolic or inflammatory stress.^132^ They provide a scalable, high‐throughput platform for drug screening and allow for CRISPR‐based mechanistic studies in patient‐derived cells.^133^

In contrast, CC mouse models offer the advantage of in vivo complexity and genetic diversity, enabling the modeling of immune–metabolic interactions, BBB dynamics, and strain‐specific susceptibility to neuroinflammation.^45^ These features are particularly useful in validating systemic drug responses, side effects, and gene–environment interactions across genetically diverse backgrounds.

**FIGURE 2:**
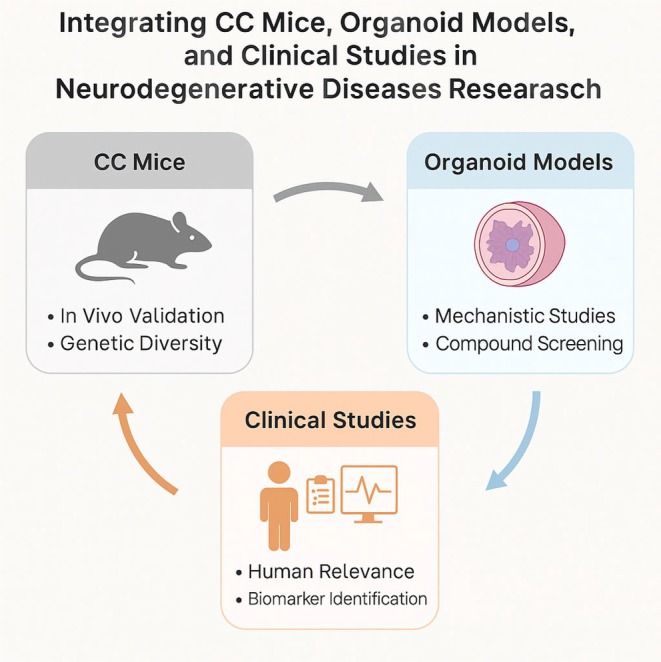
Integrative translational pipeline linking organoids, collaborative cross mice, and clinical studies in neurodegeneration research.

We propose a two‐tier research framework: (1) Organoids serve as first‐line systems for discovering candidate compounds and understanding cell‐autonomous pathways, and (2) CC mice are used to test efficacy and safety in vivo under systemic and genetic complexity. Although this model is conceptually robust, direct comparative studies between organoids and CC mice remain limited, especially in areas such as GBA modeling, neuroimmune cross talk, and sex‐based neurodegenerative differences. Future efforts should prioritize parallel experimental pipelines to harmonize findings across models and accelerate the translation of these findings. This proposed framework, illustrated in Figure 2, integrates the mechanistic precision of human organoid systems with the systemic and genetic diversity of CC mouse models. Together with clinical cohort validation, this triad forms a translational pipeline that bridges discovery, preclinical testing, and biomarker development in neurodegenerative disease research.

## 8 CONCLUSION

Inflammation, metabolic dysfunction, and autoimmunity are key contributors to neurodegenerative diseases. The CC mouse model presents an innovative method to studying how genetic diversity influences neurodegenerative risk. Subsequent investigations should prioritize precision medicine approaches that target these systemic interactions, which potentially facilitate the development of novel therapeutic strategies for AD and related dementias. The moment has arrived to treat glaucoma as an autoimmune, CNS neurodegenerative illness instead of just treating its triggers. The foundation of treatment, as with other autoimmune illnesses, is immune system control. Potential treatment approaches include both nonspecific and antigen‐targeted immunotherapies, alongside microbiome‐based interventions involving gut commensals and, potentially, microbial communities of the oral cavity and tear film.

The integration of human organoid models and CC mice represents a powerful, complementary approach for understanding neurodegeneration. Organoids enable fine‐grained, human‐specific analysis of cellular pathology and therapeutic response, whereas CC mice contextualize these findings within whole‐organism physiology and genetic variation. When combined with clinical studies, these models form a translational triad that bridges molecular discovery with therapeutic validation.

Continued refinement of both platforms—and increased efforts to link findings across them—will be essential for achieving precision medicine in dementia and related disorders.

## AUTHOR CONTRIBUTIONS

**Osayd Zohud:** Conceptualization; data curation; investigation; methodology; writing – original draft. **Iqbal M. Lone:** Conceptualization; data curation; investigation; writing – original draft. **Kareem Midlej:** Conceptualization; data curation; methodology; validation. **Fuad A. Iraqi:** Conceptualization; data curation; funding acquisition; investigation; methodology; project administration; resources; supervision; validation; visualization; writing – original draft; writing – review and editing.

## FUNDING INFORMATION

This study was supported by a core fund from Tel Aviv University.

## CONFLICT OF INTEREST STATEMENT

The authors declare no conflicts of interest. Fuad A. Iraqi is an editorial board member of *AMEM* and a coauthor of this article. To minimize bias, he was excluded from all editorial decision making related to the acceptance of this article for publication.
